# An Occlusion-Robust Feature Selection Framework in Pedestrian Detection †

**DOI:** 10.3390/s18072272

**Published:** 2018-07-13

**Authors:** Zhixin Guo, Wenzhi Liao, Yifan Xiao, Peter Veelaert, Wilfried Philips

**Affiliations:** 1 Department of Telecommunications and Information Processing, Ghent University-Interuniversitair Micro-Elektronica Centrum (IMEC), Sint-Pietersnieuwstraat 41, 9000 Gent, Belgium; Wenzhi.Liao@UGent.be (W.L.); Peter.Veelaert@UGent.be (P.V.); Wilfried.Philips@UGent.be (W.P.); 2 School of Information Science and Engineering, Shandong University, Jinan 250100, China; xiaoyifran@163.com

**Keywords:** pedestrian detection, feature selection, occlusion handling, deep learning

## Abstract

Better features have been driving the progress of pedestrian detection over the past years. However, as features become richer and higher dimensional, noise and redundancy in the feature sets become bigger problems. These problems slow down learning and can even reduce the performance of the learned model. Current solutions typically exploit dimension reduction techniques. In this paper, we propose a simple but effective feature selection framework for pedestrian detection. Moreover, we introduce occluded pedestrian samples into the training process and combine it with a new feature selection criterion, which enables improved performances for occlusion handling problems. Experimental results on the Caltech Pedestrian dataset demonstrate the efficiency of our method over the state-of-art methods, especially for the occluded pedestrians.

## 1. Introduction

Pedestrian detection plays an essential role in surveillance and security, and in computer vision for autonomous driving. Despite the large progress during the past decades [1,2,3], there is still room for improvement compared to state-of-the-art methods [4]. Research on improved features used to be one of the driving forces to improve pedestrian detectors [2], e.g., Haar [5], HOG [6], Chnftrs [7] and the popular Convolutional Neural Network (CNN) features [8,9,10]. Another more recent significant improvement is achieved by combining various feature types [7,11,12,13]. However, such richer and higher dimensional feature sets do not always lead to better detection results: the resulting representation can be very redundant and it can be more noisy than lower-dimensional feature vectors. This redundant and irrelevant information can slow down the learning process and also leads to overfitting. The same problem also exists in the popular deep learning methods.

In order to reduce the dimension of feature vectors, one solution is to create new combinations of feature attributes with linear or non-linear transformations (e.g., principal component analysis (PCA) [14], linear discriminant analysis (LDA) [15]). However, this is often computationally expensive, especially for very high dimensional data. Another approach is feature selection, which aims at selecting a subset of individual feature elements from the feature list without changing them. Depending on the feature suitability evaluation strategy, feature selection methods can be categorized into “filter” and “wrapper” types [16]. The filter method [17,18] applies a certain statistical measure as preprocessing to rank the candidate features, which is usually more computationally efficient. However, as the filter is totally independent of the classifier, it could result in non-optimal features. The wrapper method [19,20] evaluates the given subsets of features by training a classifier and testing it directly, which often leads to better selection results, but it is much slower. In this paper, we propose an “embedded structure” approach [17] in which feature selection is performed during the training process. We obtain more optimal features by analyzing the models trained in the early training stages, without increasing the overall training time.

Another challenge in pedestrian detection is occlusion, which is currently handled in the following two ways: (1) Deformable Part Models (DPM) [21] and related methods; and (2) training a set of occlusion-specific models. The DPM model has a natural advantage in handling occlusion that the detection process is separated into the detection of individual body parts, then the occluded parts can be handled individually at the decision stage. However, the DPM based method requires an accurate estimation of the visibility of different body parts [11,22], which increases the complexity of these kinds of methods. The idea of training occlusion-specific models comes from the observation that the occlusion patterns of the occluded pedestrians are limited [1], so the detection can be improved by applying unique models to distinct occlusion patterns [23]. However, it is costly to train detectors for different occlusion patterns, and it is also difficult to find a proper method to merge the results of different detectors. Inspired by the idea that some appearance characteristics in occluded pedestrian samples could be utilized to enhance the occlusion handling ability of the detector [24,25], in this paper we introduce the occluded pedestrian samples into training and propose an improved feature selection criterion, which takes the occlusion probability into account and enables the selection of more optimal features for occlusion handling.

### Contributions

The main contribution of this work is to design an occlusion-robust feature selection framework for pedestrian detection, specifically:

- Selecting features with better representative ability. During the training stage, models trained in the early stages are used to rank the strength of the features. Weak features will be removed from the candidate feature list.
- Selecting features with better occlusion handling ability. We introduce the occluded pedestrian samples into training, and propose a new feature selection criterion, which obviously improves the occlusion handling ability of the model.

In experiments, we train two models with our feature selection framework on hand-crafted channel features and learned CNN features respectively, and achieve state-of-the-art detection performance.

In addition, we also train a model with combined features (channel feature and CNN feature) and further improve the performance. By applying our feature selection framework, we provide some observations of the feature selection result.

Parts of this work have been accepted for presentation in a conference [26]. We extended [26] in the following ways: (1) instead of focusing only at the occlusion handling problem, we design a new feature selection framework which improves the general detection performance, and (2) extra experiments with the CNN features and combined features are conducted to demonstrate the effectiveness of our method.

The rest of the paper is organized as follows. We make a review of the related work in Section 2 and propose a feature selection framework to improve the detection performance in Section 3. In Section 4, a new feature selection criterion is proposed to enhance the occlusion handling ability. Experimental results are shown in Section 5, while the conclusions are drawn in Section 6.

## 2. Related Work

In this section, we will first introduce some popular feature selection methods, and then review some widely used occlusion-handling methods.

In feature selection, filter methods use specific criteria to score and rank the features, independently of the selected classifier. Therefore, filter methods act as a preprocessing step prior to classification. Many filter types have been used, including Relief-F [27,28], Laplacian [29], Fisher [30] and some improved filters such as SW-Relief-F [31]. The Relief-F [27,28] method selects features contributing to the separation of similar samples from different classes, and it is improved in SW-Relief-F [31] by assigning higher weights to samples that are close to the separating hyperplane. The Laplacian score [29] evaluates the features according to their locality preserving power, while the Fisher score [30] computes the ratio of interclass separation and intraclass variance of a feature.

Normally filter methods are not very appropriate for classification tasks because most features depend heavily on the selected classifier [32], so wrapper methods are usually preferred. The Support Vector Machine (SVM) classifier is often used to evaluate the predictive ability of features [33,34,35]. Guyon et al. [35] utilize a linear Support Vector Machine (SVM) and recursively eliminate irrelevant features to obtain the discriminate features for cancer detection. Zhang et al. [17] apply it in the driving pattern recognition. These methods [17,35] can also be categorized as embedded methods because feature selection is performed during the training of the classifier.

For occlusion handling, some researchers propose to first estimate the visibility of the body parts of pedestrians, and then handle the visible and occluded parts separately [21]. Wang et al. [11] estimate an occlusion likelihood map according to a global HOG detector, and make a final decision based only on the response of the visible parts. In [22], Ouyang et al. further explores the visibility correlations of body parts with a deep network, which supplements the detection results of the DPM model. Some additional data, such as depth and motion [36], are also used to estimate the visibility.

Another approach is to train a set of occlusion-specific detectors for different occlusion patterns [23,24,37]. Wojek et al. [37] combines the response of a full-body DPM detector and six part-based detectors to make the final decision, while Mathias et al. [23] train a more exhaustive set of ICF detectors, including 16 different occlusion patterns. In addition, some multi-person occlusion patterns are also investigated to handle occlusion in crowds. By utilizing the characteristic appearance pattern of person-person occlusion, Tang et al. [24] train a double-person detector. Ouyang et al. [25] explore this model with a probabilistic framework instead of Non-maximum Suppression (NMS) to deal with strong overlaps.

Inspired by [24,25], we propose that the occlusion informations could be utilized to improve the detection results instead of being treated as distractions. Our work differs from [24,25] in that: (1) we introduce real partial occluded samples into training instead of using manually faked person-person occlusion images; and (2) we modify the feature selection criterion by taking the occlusion probability into account, which achieves a better utilization of the occluded pedestrian samples.

## 3. The Proposed Feature Selection Framework

In recent years, methods based on the channel feature [23,38,39,40] have achieved a great success. The channel feature [7] is actually a delicate combination of 10 feature channels, including one gradient magnitude, six gradient orientations and three color channels. In this section, we aim to propose an efficient feature selection framework to remove the redundant elements of the channel feature. The feature selection results will be evaluated by testing the Aggregate Channel Feature (ACF) detector  [41] which is trained with our selected subset of features.

Instead of using the filter-based feature selection methods [27,29,30], we propose to measure the features by analyzing the trained classifier itself. This is because it is not wise to judge the strength of the features by a filter which is totally independent of the training process (see Appendix A). Our method is to judge the reliability of the features by how often they are selected. The default ACF detector contains 4096 decision trees with a maximum depth of 5. During the training of each decision stump in each tree, an exhaustive search will be applied to the candidate feature set to find the optimal one. Since the whole feature set will be repetitively searched during the training of each decision tree, we believe features that are frequently selected by the model are more important than those rarely selected. This is motivated by the idea that the frequently selected features represent more general characteristics of pedestrians, while the rarely selected ones may only suit for certain training samples and lead to overfitting.

**Figure 1:**
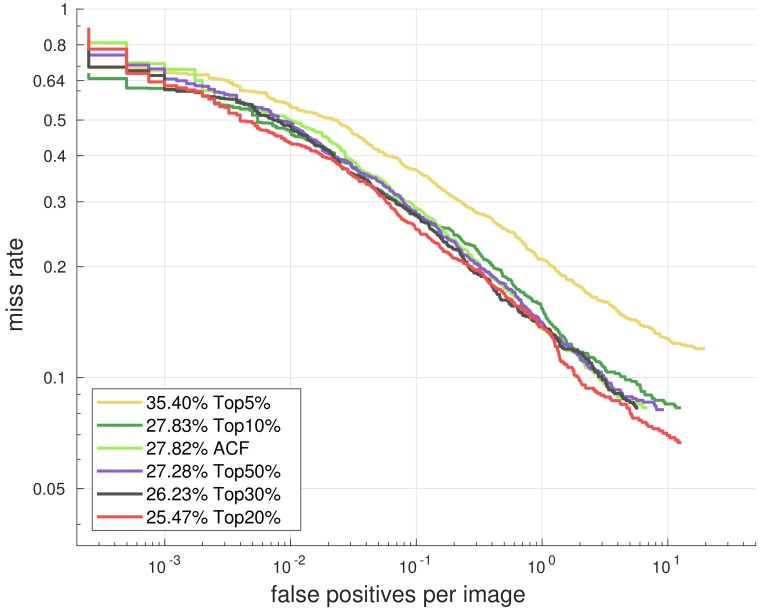
Performance of ACF detectors trained with the most frequently selected features (marked as TopN%) and full features (marked as ACF). The percentage indicates log-average Miss Rate on False Positive Per Image (FPPI) over [10^−2^, 10^0^], which is denoted as MR in the following.

Our idea is illustrated in Figure 1 and Table 1. We first train an ACF detector [41] with a complete set of 20,480 features (10 feature channels with a size of 32 × 64), and we sort the features in descending order by how many times they are selected by the detector. Then we train new models only with the top ranked 50%, 30%, 20%, 10% and 5% features (10,240, 6144, 4096, 2048 and 1024 features, respectively) and compare them with the model trained with the complete feature set.

We conclude that better performance can be reached with fewer features, and the model trained with the top 20% of most frequently selected features (4096 features) outperforms the model trained with full features (20,480 features) by about 2%. Another fact indicated in Table 1 is that there is a large difference in selection frequency between features (the Occurrence column). The 5% most frequently selected features cover 11% of the total number of selections, while the 20% most frequently selected features cover almost half of the total number of selections. So we can enhance the detector by replacing the complete candidate feature set with the top 20% features during training.

However, a downside of the approach presented so far is that we need to conduct the whole training process twice. The first training (with full features) is only used to analyze the feature selection distribution, while the second training (with the top ranked features extracted from the first model) is used to obtain the final classifier. In the next section, we will analyze the training structure of the ACF detector and propose a method to merge the two training processes into one to save time.

### 3.1. The Training Structure

**Figure 2:**
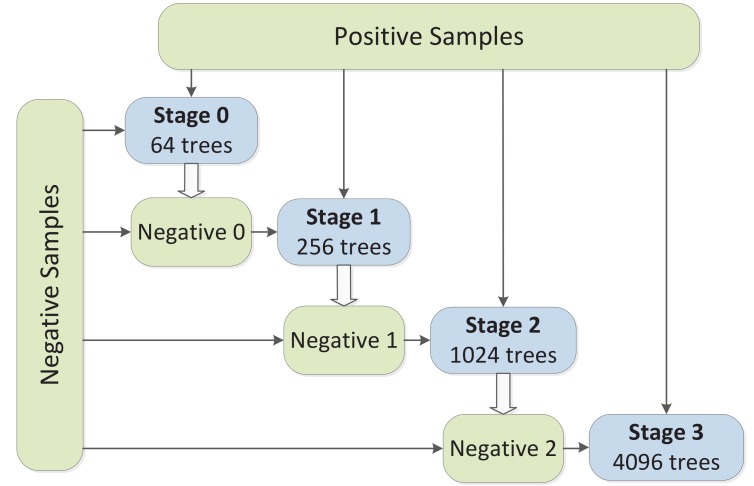
The training structure of the ACF detector, as used in [41].

Figure 2 shows the whole training process of the ACF detector which includes four training stages. The trained model in each stage has 64, 256, 1024 and 4096 decision trees, respectively. During the training phase, all positive samples (about 50,000 in the Caltech dataset [1]) are included into the training set, while negative samples are gradually added after each stage. Specifically, in the first training stage, patches randomly sampled from the non-pedestrian images act as the initial negative samples, while the added negative samples (marked as Negative 0, Negative 1 and Negative 2 in Figure 2) in the following stages come from the high-score false positive detections of the model trained in the previous stage. After each stage, the number of trees increases and the model becomes richer and enhanced. Finally, a model of 4096 trees is trained.

So far we have proposed to conduct a first training to analyze the selection frequency of features and do a second training with the top ranked features to obtain the final classifier. However, by analyzing the training structure shown in Figure 2, we observe that in each training stage a small model (with fewer trees) will be trained. So if we could predict the feature selection distribution in early stages, we can obtain the top ranked features in advance and apply them into the final stage (stage 3). In this way, a second training is not needed.

### 3.2. The Feature Selection Method

Now we calculate the feature selection distribution of the model trained in each stage (marked as model-*n*, *n* denotes the stage number 0, 1, …, 3, see Figure 2) and sort them in descending order. We have already shown in Table 1 that the top ranked 20% features of model-3 (same with the final classifier) can act as better candidate features in training, so we define these 4096 features as the target features. If the top ranked features of model-0,1,2 can include most of the target features, we can use them as the candidate features in stage 3 and expect a similar performance improvement achieved by the target features in Table 1.

**Figure 3:**
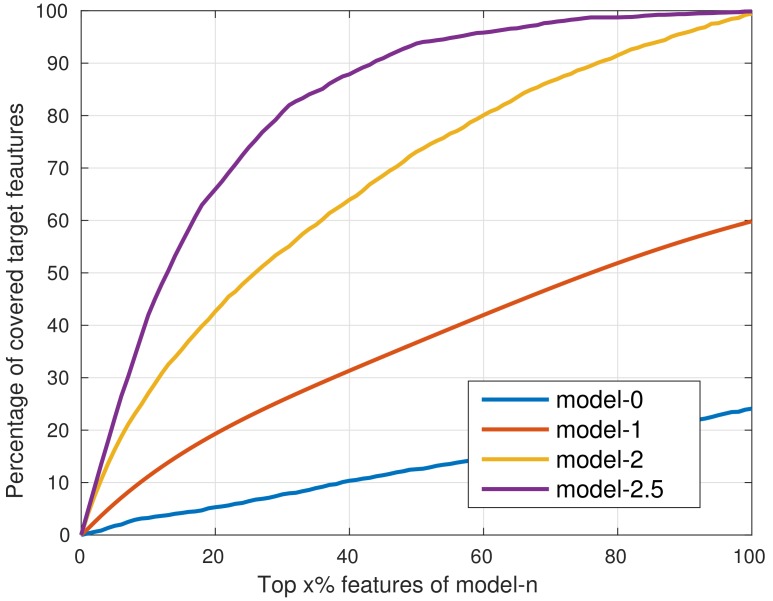
Comparison of the most frequently selected features extracted from the early trained models with the target features. The most frequently selected 50% features of the model-2.5 has covered almost 95% of the target features.

In Figure 3, we compare the top ranked features of model-*n* with the target features. The horizontal axis represents the percentage of the top ranked features of model-*n*, while the vertical axis denotes the percentage of target features that are included by the top ranked x% features of model-*n*. Unsurprisingly, Figure 3 shows that as the training proceeds, the top ranked features of later trained models cover the target features better, while the top ranked 50% features of the half-trained model in stage 3 (the first 2048 trees, marked as model-2.5) have covered almost 95% of the target features. So during the last training stage (stage 3), we can first train 2048 trees to analyze the feature selection distribution, and then use the top ranked 50% features to retrain the final model of 4096 trees. In this way, most of the target features are included by the candidate feature list during the last training stage. The detailed training process is shown in Algorithm 1.

**Algorithm 1** Modified Training Process
**Input:** positive samples Pos, negative samples $Neg_{0}$

**Initialize:**N=3, Tnum={64,256,1024,4096}, $F=\{F_{1},F_{2},\cdots ,F_{20480}\}$


1:**for** stage i=0,1,2 **do**2:   Train $Tnum_{i}$ trees with Pos and $Neg_{i}$, over the candidate feature list *F*, obtain $model_{i}$3:   Conduct detection with $model_{i}$ on non-pedestrian images, obtain hard negative samples $Neg_{i+1}$4:Train $Tnum_{N}/2$ trees with Pos and $Neg_{N}$, over the candidate feature list *F*, obtain $model_{N}^{{}^{\prime }}$5:Calculate and rank the most selected 50% features, update the candidate feature list with $F^{top50}$6:Retrain $Tnum_{N}$ trees with Pos and $Neg_{N}$, over the candidate feature list $F^{top50}$, obtain $model_{N}$



**Output:** $model_{N}$



Another advantage of this method is that the overall training time will not increase. In the last training stage, we define the original training time as *T*. Since the final 4096 trees are trained over a halved candidate feature list, the training time is also reduced to about *T*/2. Adding up the first 2048 trees trained for analyzing, the total training time of the last stage in our method is *T*/2 + *T*/2 = *T*.

## 4. Occlusion Handling

In principle, we can extract some characteristic occlusion appearance features from the occluded pedestrian samples to assist the detection of occluded pedestrians. However, we have observed that most existing approaches intentionally avoid using such samples during the training stage, because unreliable information will probably be introduced into training and this results in a decline in general performance. As is shown in Table 2, we train two ACF detectors [41] with and without the occluded pedestrian samples. In the Reasonable test case, which only contains fully visible or less than 35 percent occluded pedestrians, the detector trained with occluded samples shows a performance decline. However, for only occluded pedestrians, an improvement is achieved by training with occluded samples.

Table 2 illustrates that introducing occluded samples into training makes sense, at least for the detection of occluded pedestrians. On this basis, we will propose a new feature selection criterion to enhance the occlusion-handling ability of the detector, but without lowering the *general* detection performance. The occlusion probability will be considered in the proposed criterion, which better utilize the information of the occluded training samples.

### 4.1. Feature Selection Criterion in ACF

In order to explain how occlusion probability is included in our proposed criterion, we briefly review the training mechanism employed in ACF. It employs a boosting structure that greedily minimizes a loss function for the final decision rule $F\left(x\right)=\sum_{t}\alpha_{t}f_{t}\left(x\right)$, where the final classifier F(x) is a weighted sum of *t* weak classifiers $f_{t}\left(x\right)$. $x\in R^{K}$ denotes the feature vector while $\alpha_{t}$ indicates the weight of each weak classifier. At each iteration *t*, a decision tree $f_{t}\left(x\right)$ is trained and its weight $\alpha_{t}$ is optimized.

A decision tree f(x) is composed of a stump $h_{j}\left(x\right)$ at every non-leaf node *j*. The tree is grown from the root node by recursively learning a stump at a time, which produce a binary decision:

(1) h(x)= p sign(x[k] − τ)

where x[k] denotes the *k*-th feature of the sample x, *p* and τ are the polarity element {±1} and threshold for feature *k*. During the learning of a stump, the goal is to optimize *k*, *p* and τ, which minimize the classification error:

(2) $$\epsilon =\sum \omega_{i}1_{\left\{h\left(x_{i}\right)\neq y_{i}\right\}}$$

where $1_{\left\{\ldots \right\}}$ is the indicator operator. $h(x_{i})$ and $y_{i}$ indicate the classification result and the true class of the input feature $x_{i}$, respectively. $\omega_{i}$ is the weight of each sample, which is updated after each iteration *t* (the weight of the misclassified samples will be increased).

Specifically, by combining Equations (1) and (2), the classification error can be rewritten as:

(3) $$\epsilon_{\left(k\right)}=\sum_{x_{i}\left[k\right]\leq \tau }\omega_{i}1_{\left\{y_{i}=+p\right\}}+\sum_{x_{i}\left[k\right]>\tau }\omega_{i}1_{\left\{y_{i}=-p\right\}}$$

So the feature selection is conducted by selecting the most optimal feature *k* and its corresponding parameters *p* and τ that minimize the classification error: $\left\{k,p,\tau \right\}=argmin\;\epsilon_{\left(k\right)}$.

However, when we introduce occluded pedestrian samples into training, this feature selection criterion concerning only the classification error will not be sufficient. The selection is susceptible to noisy image patches, and in particular, to the occluded regions of the pedestrian samples. For example, if a feature in the occluded region of a pedestrian is selected, it harms the learning of pedestrian characteristics because it is very different from pedestrian but is labeled as a pedestrian. In the next we will propose a more robust feature selection criterion under the occlusion occasions.

### 4.2. Occlusion-Robust Feature Selection Criterion

The key insight of our method is to help the classifier avoid selecting features in the occluded regions of the training samples, and therefore improve the detection performance.

**Figure 4:**
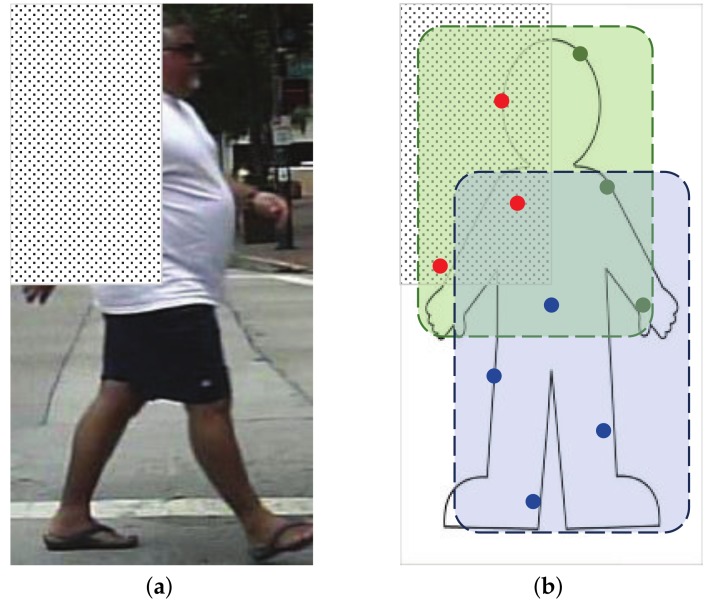
(**a**) Pedestrian samples are manually occluded in the top-left corner with non-person image patches randomly cut from negative samples. (**b**) Some features in the occlusion region (marked as red) are selected by weak classifiers but they are not reliable.

To demonstrate this idea conceptually, we assume the simplest scenario in which all the training samples are occluded in the same region, see Figure 4. We manually occluded all the training samples in the top-left corner in Figure 4a. There are two weak classifiers in Figure 4b, which are marked as blue and green region, respectively. Each weak classifier will select several pixels from the feature map. Since the occluded region is known to be in the top-left corner, we are sure that features marked as red points are unreliable. In Appendix B, we successfully enhanced the trained detector by preventing the selection of features from the occluded region.

Of course, in a realistic training process, any part of a pedestrian can be occluded, which makes it necessary to investigate the occlusion probability of each feature. So we propose to create occlusion distribution maps and a new feature selection criterion in this section.

#### 4.2.1. Occlusion Distribution Map

**Figure 5:**
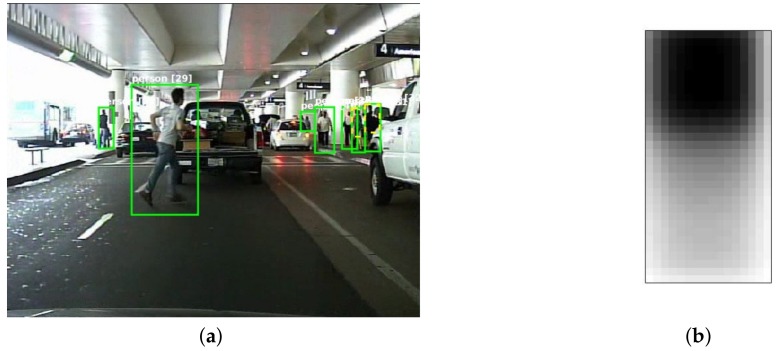
Calculate the occlusion distribution with partially occluded pedestrians. (**a**) A picture with partially occluded pedestrians from the Caltech pedestrian dataset; (**b**) Occlusion distribution map of the whole partially occluded pedestrian samples. The brighter region indicates higher probability of being occluded.

In the Caltech Pedestrian dataset [1], each partially occluded pedestrian is annotated with two bounding boxes, which indicate the full body (green boxes) and the visible part (yellow boxes), respectively (see Figure 5a). First we create a binary occlusion mask map for each partially occluded pedestrian sample. By averaging all the mask maps of the occluded samples, we obtain an occlusion distribution map in Figure 5b. This map shows that the occlusion distribution is not uniform: normally the lower part of a pedestrian has a higher probability of being occluded, which conforms to the common sense that the head region is more likely to be visible in real world, because most obstacles are located on the ground.

Note that this map (Figure 5b) is not what we will use in training to get the occlusion probabilities of features, as when training a weak classifier (decision tree), the training samples will be separated and delivered to different nodes at each stump (as explained in Equation (1)). So we will calculate a new occlusion map with the existing training samples at each stump and apply it to the new feature selection criterion.

#### 4.2.2. New Feature Selection Criterion

Instead of judging a feature only by its classification error, now we propose a new feature selection criterion which takes into account the occlusion probability. The key principle is: if two features have very similar classification errors, we prefer the one with lower occlusion probability. Therefore, a new cost function $\epsilon_{\left(k\right)}^{\prime }$ (when feature *k* is selected) can be defined:

(4) $$\epsilon_{\left(k\right)}^{\prime }=\epsilon_{\left(k\right)}\cdot \left(1+\gamma_{\left(k\right)}\right)$$

(5) $$\gamma_{\left(k\right)}=\theta N_{\left(k\right)}^{occ}/N^{pos}$$

where $\epsilon_{\left(k\right)}$ indicates the original classification error defined in Equation (3), $\gamma_{\left(k\right)}$ represents the occlusion cost coefficient of feature *k*, which is determined by its occlusion probability $N_{\left(k\right)}^{occ}/N^{pos}$. Here $N^{pos}$ indicates the number of positive samples in the current stump, while $N_{\left(k\right)}^{occ}$ indicates the number of samples which have an occlusion in the location of feature *k*. The occlusion probability $N_{\left(k\right)}^{occ}/N^{pos}$ can be obtained from the occlusion distribution map calculated in the current stump (see Section 4.2.1). In order to balance the weight of classification error and occlusion probability, we add a constant weight θ in $\gamma_{\left(k\right)}$ which is much smaller than 1 (we use an experience value 0.1 in this paper) to ensure the priority of the discriminative ability of a feature. Under this new criterion, the classifier will prefer a feature with low occlusion probability to a feature that is barely better but has much higher occlusion probability. Finally the selection is made by minimizing the new cost function:

(6) $$k\;=\;argmin\;\epsilon_{\left(k\right)}^{\prime }$$

**Figure 6:**
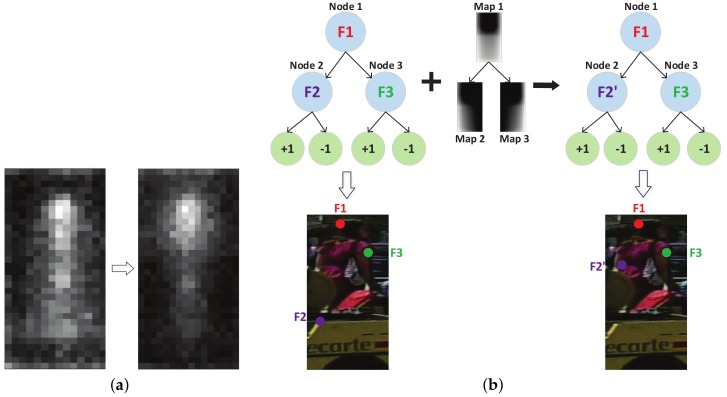
Occlusion-robust feature selection criterion. (**a**) By including the occlusion distribution map, feature selection will be more biased to the low occlusion probability regions, as shown in the right; (**b**) Feature F2 selected by the original training procedure are replaced by F2’ according to the occlusion distribution map in node 2.

In Figure 6a, the new model trained under the proposed criterion is more biased to select the features from low occlusion probability regions (for example, the head region). Figure 6b shows how our proposed method works during the training of a decision tree classifier. Under the original feature selection criterion (Equation (3)), feature F1, F2 and F3 will be selected by the nodes of the classifier. Then we calculate the occlusion distribution map on each node and employ Equations (4)–(6) to select more reliable features. For example, feature F2 is replaced by a more reliable feature F2’ which is less likely to be occluded according to the occlusion distribution map at that node.

## 5. Experiments

In order to demonstrate the effectiveness of our method, we conduct two experiments using hand-crafted channel features and learned CNN features respectively. Our methods are evaluated on the Caltech Pedestrian Dataset [1], which includes both Reasonable and Occlusion test cases. In the Reasonable subset, only fully visible or less than 35 percent occluded pedestrians are under evaluation. The partial and heavy occlusion subsets include 1–35 and 35–80 percent occluded pedestrians, respectively. The log-average miss rate is used to evaluate the detection performance of the detectors, and a smaller value indicates a better performance.

### 5.1. Experiments Using Channel Features

In this section we will first explore the potential of the ACF model, and then apply our proposed feature selection methods on the optimal trained models and compare them with the state-of-the-art channel feature based methods. We double the default model size to 41 × 100 pixels, which results in a richer feature of 32 × 64 pixels per channel. Positive samples in most experiments are obtained by sampling the Caltech video data with a skipping step equal to 10, while more dense sampling is also conducted in some cases.

**Figure 7:**
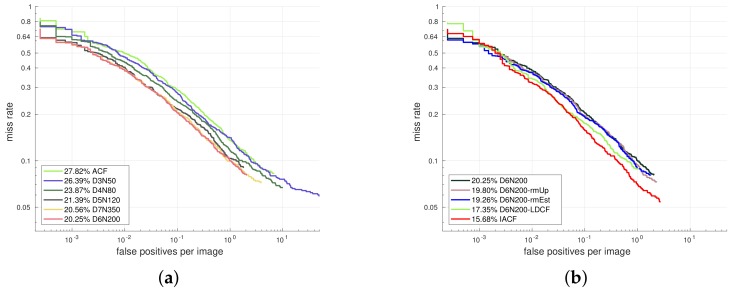
Optimal training of the ACF model.

At first we investigate the optimal training parameters by gradually increasing the maximum depth of the model. For larger model capacity (deeper trees), more training samples are needed to explore the full potential of the model. We start our experiment with depth 3 and gradually increase the depth to 7 with more training samples (see Figure 7a). At the start, we train a maximum depth 3 model with 50,000 negative samples, named as D3N50 (D indicates depth while N indicates the number of negative samples). In order to find the optimal data size under certain depth, we conduct a series of experiments with different number of samples. When additional data does not lead to an performance improvement, the model is considered to be saturated. In this way, models with a tree depth from 3 to 7 are evaluated with 50,000, 80,000, 120,000, 200,000 and 350,000 negative samples, respectively. For depth 6 and 7, more dense sampling with a skipping step of 4 is employed to enlarge the number of positive samples. In Figure 7a a quick performance saturation occurs: when the tree depth reaches 6, larger model capacity or additional data seems to have little help, so we fix D6N200 as our baseline setting and further improve it in Figure 7b.

**Figure 8:**
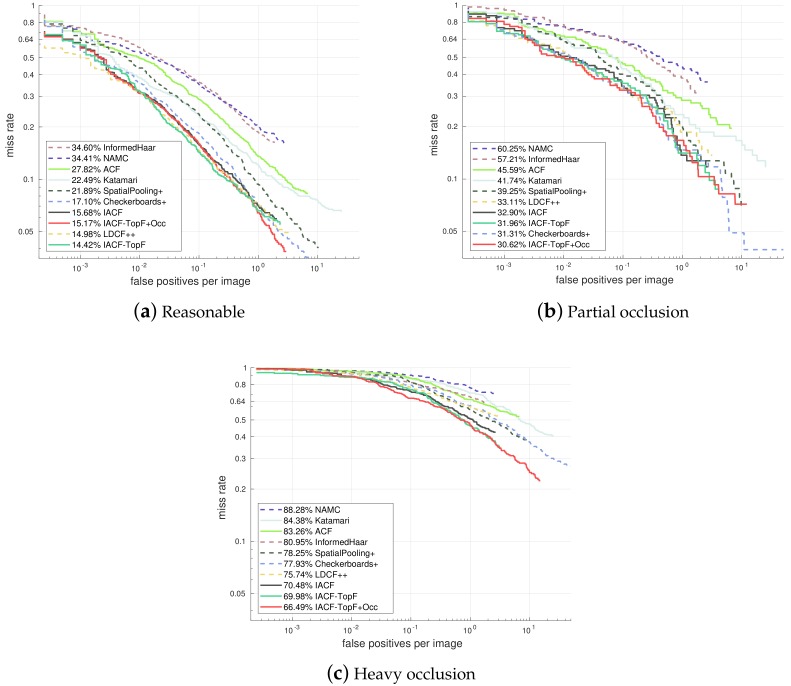
Comparison with the state-of-the-art channel feature based methods.

At first we employ feature decorrelation filtering [40] and obtain D6N200-LDCF, then we remove feature estimation during the feature calculating process as suggested in [42] and get D6N200-rmEst. Furthermore, we utilize some prior knowledge from the real world by removing candidate windows from the upper 1/3 of the image, which obviously reduces the calculation and avoids some false positive detections in the non-pedestrian regions (D6N200-rmUp). With all the above modifications, we obtain our optimal trained detector named IACF (improved ACF), which is comparable with the state-of-the-art channel feature based method LDCF++ [42], see Figure 8a.

Now we apply our proposed feature selection methods to IACF and compare it with the state-of-the-art channel feature based methods. In the final training stage, the model is trained only with the most frequently selected 50% features of the model-2.5 (see Algorithm 1), and we get IACF-TopF. Then in order to improve the occlusion handling ability, we further introduce our proposed feature selection criterion into the model and get IACF-TopF-Occ.

In the commonly used Reasonable case (Figure 8a), our IACF-TopF shows an obvious improvement from 15.68% to 14.42% (log-average Miss Rate on False Positive Per Image (FPPI) over [10^−2^, 10^0^]) after we apply our proposed feature selection method, and this is also the best result compared with some state-of-the-art channel feature based method. Although there is a slight performance decline of our IACF-TopF-Occ model, it is still comparable with the state-of-the-art detectors. In addition, our IACF-TopF-Occ achieves a better occlusion handling performance.

In the partial occlusion case (Figure 8b), our IACF-TopF-Occ model obtains the best result of 30.62%. More impressive results appear in the heavy occlusion case (Figure 8c), which achieves a significant improvement (from 75.74% to 66.49%) over the state-of-the-art channel feature based model LDCF++, while the efficiency remains the same (only the selected features and thresholds are changed during detection).

### 5.2. Experiments Using CNN Features

Recently, methods based on CNN features [10,13,43,44,45,46] have achieved the state-of-the-art performance in pedestrian detection. So we also apply our feature selection method on CNN features to obtain a further improvement.

We adopt the RPN+BF [10] framework, which includes an Region Proposal Network (RPN) [47] to generate candidate bounding boxes and a Boosted Forest (BF) classifier. The RPN is built on the top of the Conv5_3 layer of the VGG-16 net [48], and an ROI pooling [49] is adopted to extract features of fixed length (7 × 7) from the candidate regions. The BF classifier is trained in six bootstrapping stages with 64, 128, 256, 512, 1024 and 2048 decision trees respectively. In RPN+BF, a combination of features from Conv3_3 and Conv4_3 is used in training, which makes a feature size of (256 + 512) × 7 × 7 = 37,632. We employ our proposed feature selection method by calculating the selection frequency of the features in stage 4 (1024 trees), then in the last stage (2048 trees), the final model is trained only with the top ranked 20% features (6527 features).

**Figure 9:**
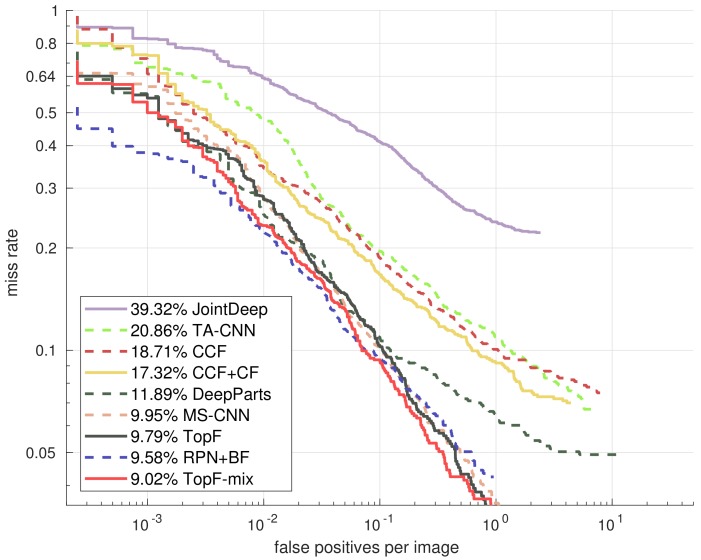
Comparison with state-of-the-art CNN based methods in reasonable subsets.

We use TopF to mark the detector trained with our proposed method and compare it with the state-of-the-art CNN feature based methods in Figure 9. Note that we achieve an average miss rate of 10.32% with our implementation of RPN+BF [10], which is not as good as the published results (9.58%), but with our proposed method, the TopF reaches a comparable performance of 9.79%. We further enhance the detector by training combined features of hand-crafted channel features and learned CNN features, and then we also apply our feature selection method with the mixed features and get the TopF-mix, which has a candidate feature size of (37,632 + 64 × 32 × 10) × 20% = 11,623. Figure 9 shows that our TopF-mix model achieves a further improvement from 9.79% to 9.02%.

We also make an additional investigation of the mixed features. We train a new model with the full candidate features (37,632 + 64 × 32 × 10 = 58,112) and sort them in descending order by how many times they are selected by the model, then we analyze the composition of the two feature types. In Table 3, we find the top ranked features are mostly selected from the CNN features. For example, the CNN feature accounts for 65% of the full 58,112 features, but when we focus only on the most selected 50% features, the proportion increases to 74%. In our TopF-mix model trained with the most selected 20% features, CNN features accounts for almost 80% of the feature mixtures. This observation proves that the learned CNN features do have better representative ability compared with the traditional hand-crafted channel features, and there is still room for improvement. Combining multiple feature types remains an effective way to enhance the detection performance of the detectors.

## 6. Conclusions

In this work, we have proposed a robust feature selection framework for pedestrian detection. At first, we propose to evaluate the representative ability of the features by its selection frequency in the early training stages, then the seldomly selected features are regarded as redundant features and are removed from the candidate feature list in the final training stage. It achieves a better detection performance in general cases. Then, in order to handle the occlusion problems, we propose an alternating feature selection criterion which takes into account the occlusion probability of each feature. The effectiveness of our proposed framework is confirmed by the comparison experiments with the state-of-the-art methods, which is conducted both on the hand-crafted channel features and learned CNN features. In the end, we provide an observation on the feature selection results from our method. It confirms the power of CNN features, and also points out that the combination of multiple types of features still remains a good improvement approach.

## Figures and Tables

**Table 1:** ACF detectors trained with the most frequently selected features. The 20% most frequently selected features cover 48% of the selection times and lead to an obvious performance improvement.

Percent	Num	Occurrence	MR
100%	20,480	100%	27.82%
Top50%	10,240	76%	27.28%
Top30%	6144	61%	26.23%
**Top20%**	**4096**	**48%**	**25.47%**
Top10%	2048	24%	27.83%
Top5%	1024	11%	35.40%

**Table 2:** Detection performance of models trained with and without occluded samples.

	Reasonable	Partial Occlusion	Heavy Occlusion
ACF (without occ)	**27.04%**	48.53%	88.58%
ACF (with occ)	29.87%	**48.12%**	**82.73%**

**Table 3:** Composition of the combined features.

	CNN Feature	Channel Feature
Full	65%	35%
Top50%	74%	26%
Top20%	79%	21%
Top10%	82%	18%

**Figure A1:**
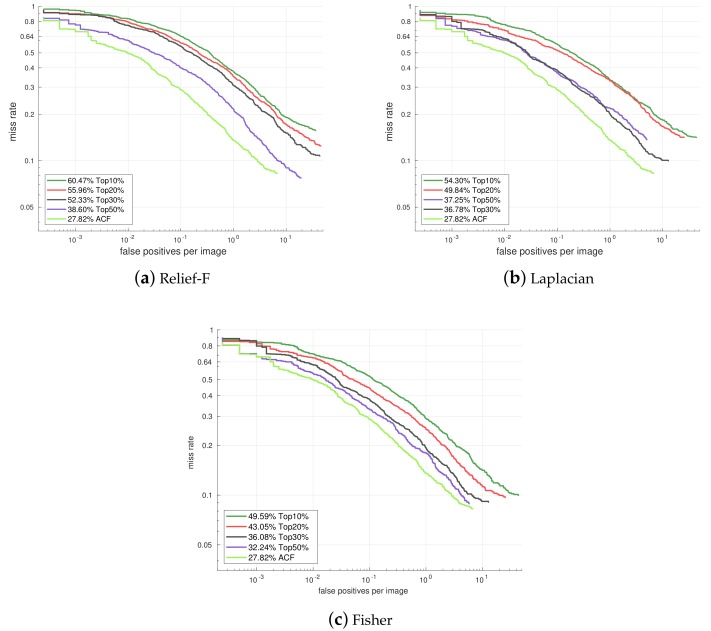
Performance of the filter-based feature selection methods.

**Figure A2:**
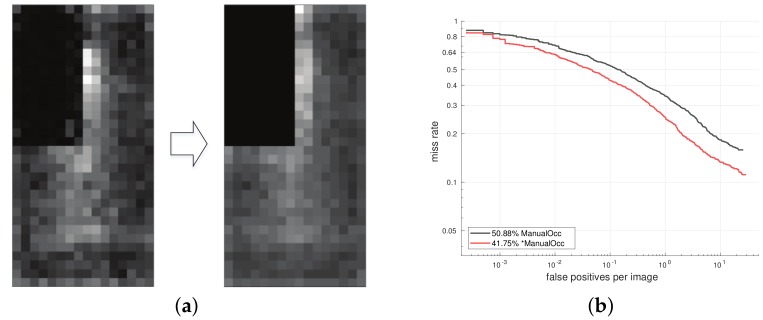
A simple situation that occlusion region is fixed. (**a**) The detector is forced to only select features from the unoccluded regions during training. (**b**) The detector trained with biased feature selection strategy shows an improved performance.

## Appendix A

To test the filter-based feature selection methods (Relief-F [27], Laplacian [29] and Fisher [30]), we adopt the implementation in [50], while the ACF [41] detector is used to evaluate the selection result. Here we apply the default ACF settings in [41], except that we double the model size to 41 × 100 pixels, which results in a feature size of 32 × 64 pixels per channel, so the training will be done over 32 × 64 × 10 = 20,480 candidate features. The Caltech pedestrian dataset [1] is used in training (sets S0–S5) and testing (sets S6–S10). Each method is tested in two steps:

- Score and rank the candidate features with the feature selection method. Then we save the top ranked 10%, 20%, 30% and 50% features (2048, 4096, 6144 and 10,240 features respectively) for evaluation.
- Train ACF models only with the top ranked features, and compare them with another model trained with full features.

Figure A1 and Table A1 shows the tested feature selection methods result in a dramatic performance decline, which demonstrate that using a specific criterion to select features before training will harm the predictive ability of the trained classifier.

**Table A1:** Performance of the filter-based feature selection methods.

Methods	Full	Top50%	Top30%	Top20%	Top10%
Relief-F	**27.82%**	38.60%	52.33%	55.96%	60.47%
Laplacian	**27.82%**	37.25%	36.78%	49.84%	54.30%
Fisher	**27.82%**	32.24%	36.08%	43.05%	49.59%

## Appendix B

To simplify the occlusion problem, we assume a simple situation that all samples has a fixed occlusion region, which can be handled by controlling the feature selection procedure during the training. Default ACF [41] settings are applied in this section.

We manually occluded all the training samples with non-pedestrian image patches in the top-left corner which covers 1/4 of the image (see Figure 4a). The non-pedestrian image patches were randomly cut from the negative training samples. By analyzing the feature selection distribution of the trained model, we find most of the selected features are from the unoccluded region, but a few features in the top-left corner are still selected during training (see Figure A2a). So we train a new model which is forced to select features in unoccluded regions, and an obvious improvement is shown in Figure A2b.
